# Supplementary material for: HER2-Low Luminal Breast Carcinoma Is Not a Homogenous Clinicopathological and Molecular Entity
Source: Cancers (Basel). 2024 May 25;16(11):2009. doi: 10.3390/cancers16112009 (PMC11171142; doi:10.3390/cancers16112009)
Supplement: Supplementary file 1 [file cancers-16-02009-s001.zip › Supplementary Data.pdf]

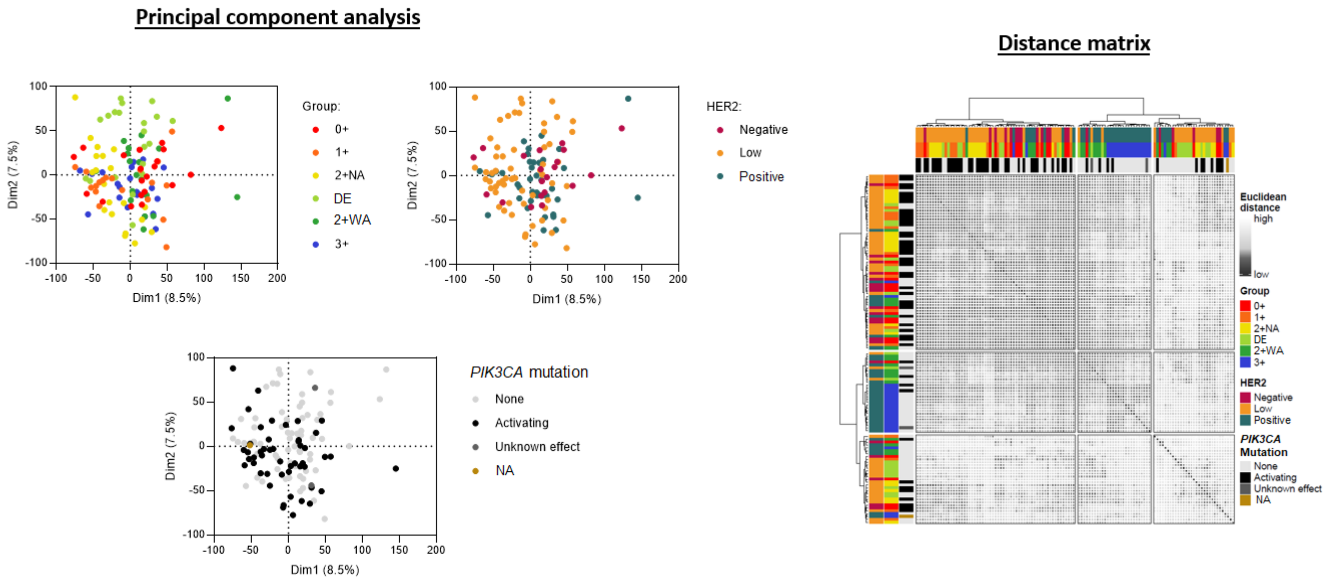

Figure S1. Whole transcriptome analysis of hormone receptor-positive breast carcinomas A comparison of three IHC HER2 tumor groups (HER2-low (H2L), HER2-negative, and HER2-positive), then six ASCO/CAP HER2 groups, and finally of tumors stratified by the presence of an activating *PIK3CA* mutation. **A)** Principal component analysis. **B)** Heatmap for the three pathways.

2+NA: non-amplified tumors; 2+WA: weakly-amplified tumors; ASCO/CAP: American Society of Clinical Oncology / College of American Pathologists; DE: HER2 double-equivocal tumors; NA: data not available

Table S1. Immunohistochemical analysis of protein expression in breast tumors: antibodies, protocol and result interpretation details

**Analyzed protein**	**Antibody Clone**	**Antigen retrieval and detection kit**	**Staining pattern**	**Result interpretation**
**ER**	Clone SP1, prediluted<br>(Roche™ (Ventana))	TBE pH 8.4, Ultraview DAB	Nuclear	Positive: ≥ 10% stained cells with any intensity score (1 through 3), in the infiltrative component only<br><br>Negative: less than 1% tumor cells stained<br>(tumors with scores between 1 and 9% were not included in the study)<br><br>As currently recommended [4]
**PR**	Clone 1E2, prediluted<br>(Roche™ (Ventana))	TBE pH 8.4, Ultraview DAB	Nuclear	Positive: ≥ 10% stained cells (intensity 1 through 3) in the infiltrative component only<br><br>Negative: less than 1% tumor cells stained<br>(tumors with scores between 1 and 9% were not included in the study)<br><br>As currently recommended [4]
**Ki67**	Clone MIB-1, dilution 1/50<br>(Agilent™ (Dako))	TBE pH 8.4, Ultraview DAB	Nuclear	Percentage (%) of stained tumor cells

ER: estrogen receptor; PR: progesterone receptor; TBE: Tris-borate-EDTA

Table S2. Pathogenic gene variants identified in HER2-low (H2L), HER2-negative, and HER2-positive breast carcinomas by DNA sequencing.

Gene	Variant	Consequence	H2L	HER2-negative	HER2-positive
PIK3CA	p.(Arg108His)	Missense	0	0	0
	p.(Glu110del)	In-frame deletion	0	0	1
	p.(Glu365Lys)	Missense	0	0	1
	p.(Glu453Lys)	Missense	0	0	0
	p.(Glu542Gln)	Missense	1	0	0
	p.(Glu542Lys)	Missense	6	0	1
	p.(Glu545Lys)	Missense	3	3	1
	p.(Gly1049Arg)	Missense	1	0	0
	p.(Gly118Asp)	Missense	2	0	0
	p.(His1047Arg)	Missense	9	3	1
	p.(His1047Leu)	Missense	2	0	1
	p.(His450_Leu455del)	In-frame deletion	1	0	0
	p.(Met1043Val)	Missense	0	1	0
	p.(Val105_Arg108del)	In-frame deletion	1	0	0
	p.(Val344Met)	Missense	0	1	0
	p.(Val346Glu)	Missense	1	0	0
	p.(Val448_Gly451del)	In-frame deletion	1	0	0
AKT1	p.(Glu17Lys)	Missense	5	0	0
PTEN	p.(Arg378fs)	Deletion - Frameshift	0	0	1
	p.(Asp252Gly)	Missense	1	0	0
TP53	c.920-2A>C	SNV intronic	0	0	1
	p.(Ser127Phe)	Missense	0	0	1
	p.(Arg213Arg)	Synonymous	0	0	1
	p.(Met133Lys)	Missense	0	0	1
	p.(Tyr236Asp)	Missense	0	0	1
	p.(Gly302fs)	Frameshift	0	0	1
	p.(Val272Met)	Missense	0	0	1
	p.(Arg337Cys)	Missense	0	0	1
	p.(Gln104*)	Nonsense	1	0	0
	p.(Ser185fs)	-	0	0	1
	p.(Tyr220His)	Missense	0	0	1
	p.(His179Arg)	Missense	0	0	1
	p.(His233del)	In-frame deletion	0	0	1
	p.(Thr125=)	Synonymous	1	0	0
	p.(Lys120Ter)/p.(Arg283Cys)	Nonsense/Missense	1	0	0
BRCA2	p.(Ser1667*)	-	0	0	1
	p.(Val1283fs)	Frameshift	1	0	0
	c.8755-1G>T	Splice acceptor	1	0	0
	Tyr2601Trpfs*46	Splice donor protein truncated	0	0	1
	p.(Lys2077ArgfsTer4)	In-frame deletion	1	0	0
ARID1A	p.(Gln200*) / p.(Pro225fs)	-	0	0	1
BRCA1, PALB2, KRAS, NRAS, BRAF	No variant found				

SNV: single nucleotide variant
